# Validation of Selected Head and Neck Cancer Prognostic Markers from the Pathology Atlas in an Oral Tongue Cancer Cohort

**DOI:** 10.3390/cancers13102387

**Published:** 2021-05-14

**Authors:** Anna Maria Wirsing, Inger-Heidi Bjerkli, Sonja Eriksson Steigen, Oddveig Rikardsen, Synnøve Norvoll Magnussen, Beate Hegge, Marit Seppola, Lars Uhlin-Hansen, Elin Hadler-Olsen

**Affiliations:** 1 Department of Medical Biology, Faculty of Health Sciences, UiT The Arctic University of Norway, 9037 Tromsø, Norway; anna.wirsing@uit.no (A.M.W.); Inger-heidi.bjerkli@unn.no (I.-H.B.); sonja.eriksson.steigen@unn.no (S.E.S.); oddveig.rikardsen@unn.no (O.R.); synnove.magnussen@uit.no (S.N.M.); beate.hegge@uit.no (B.H.); Marit.seppola@uit.no (M.S.); Lars.uhlin-hansen@uit.no (L.U.-H.); 2 Department of Otorhinolaryngology, University Hospital of North Norway, 9038 Tromsø, Norway; 3 Department of Clinical Pathology, University Hospital of North Norway, 9038 Tromsø, Norway; 4 The Public Dental Health Service Competence Centre of Northern Norway, 9019 Tromsø, Norway

**Keywords:** prognostic marker, the pathology atlas, the cancer genome atlas, oral tongue squamous cell carcinoma, Calmodulin-like 5 (CALML5)

## Abstract

**Simple Summary:**

Prognostic markers are used to predict the aggressiveness of a cancer and to help decide the best treatment for individual patients. Despite intense research, reliable prognostic markers for oral cancer are still few. The aim of the present study was to validate selected prognostic markers for head and neck cancer identified by unbiased approaches in oral tongue cancer, a specific subsite of head and neck cancer. From a list of 790 markers, we selected three based on reported prognostic value as well as expression pattern and availability of validated antibodies. These were analyzed on transcriptional and protein level in a cohort of 121 oral tongue cancers. Only one of the markers showed significant prognostic value when controlling for established prognostic parameters. Our study highlights the need to evaluate prognostic markers in homogeneous groups of cancers and to control for established prognostic parameters.

**Abstract:**

The Pathology Atlas is an open-access database that reports the prognostic value of protein-coding transcripts in 17 cancers, including head and neck cancer. However, cancers of the various head and neck anatomical sites are specific biological entities. Thus, the aim of the present study was to validate promising prognostic markers for head and neck cancer reported in the Pathology Atlas in oral tongue squamous cell carcinoma (OTSCC). We selected three promising markers from the Pathology Atlas (*CALML5*, *CD59*, *LIMA1*), and analyzed their prognostic value in a Norwegian OTSCC cohort comprising 121 patients. We correlated target protein and mRNA expression in formalin-fixed, paraffin-embedded cancer tissue to five-year disease-specific survival (DSS) in univariate and multivariate analyses. Protein expression of *CALML5* and *LIMA1* were significantly associated with five-year DSS in the OTSCC cohort in univariate analyses (*p* = 0.016 and *p* = 0.043, respectively). In multivariate analyses, lymph node metastases, tumor differentiation, and *CALML5* were independent prognosticators. The prognostic role of the other selected markers for head and neck cancer patients identified through unbiased approaches could not be validated in our OTSCC cohort. This underlines the need for subsite-specific analyses for head and neck cancer.

## 1. Introduction

The search for prognostic and predictive biomarkers has been a focus for cancer research over the past decades. The overall aim is to provide tailored treatments with a better and more predictable outcome. This may increase survival by identifying those who will benefit from specific treatments, but also reduce unnecessary side effects and costs by avoiding treatment of patients who will not profit from it. For some cancer types, such as breast cancer, expression of certain genes or proteins form the basis for subclassification of tumors, choice of treatment, and prognostication [1]. For other cancers reliable biomarkers are still missing, and the treatment is based on traditional parameters, such as the tumor size (T status), and the presence and extent of lymph node metastases (N status) and distant metastases (M status).

Traditionally, the search for prognostic markers has been hypothesis-driven, based on knowledge of established roles of molecules in cancer-associated processes, such as proliferation, cell death, invasion, metastases, angiogenesis, or inflammation. However, many molecules may have multiple functions, and functions that are not well characterized, which may explain the limited success of this approach. To avoid being restricted by our imagination and the current state of knowledge, so-called unbiased searches for biomarkers have become increasingly popular over the past decade. This approach takes advantage of high-throughput methods such as RNA or DNA sequencing, proteomics, or metabolomics, where a large proportion of target molecules in a sample are analyzed simultaneously and correlated to the outcome of interest.

Recently, the Human Protein Atlas (HPA) program published the Pathology Atlas. This comprehensive project presents the prognostic value of all protein-coding transcripts in 17 major human cancers [2], and is based on more than 900,000 survival plots from transcriptional and clinical data of the Cancer Genome Atlas (TCGA). Each of the 17 major cancers in the TCGA includes various subgroups of cancer that may have distinct biological profiles and clinical behavior. Head and neck squamous cell carcinomas (HNSCC) are among the cancers included in this work. HNSCC comprise cancers in the oral cavity, the nasopharynx, the sinuses, the oropharynx, the hypopharynx, and the larynx, of which oral cavity cancer is the most common [3]. The most important shared risk factors for HNSCC are high alcohol and tobacco use [4]. High-risk human papilloma virus (HPV) and Epstein–Barr virus are considered important risk factors for oropharyngeal and nasopharyngeal cancers, respectively, but no convincing association has been found between these viruses and oral cancer [5,6]. Genome-wide characterization of TCGA HNSCC revealed a wide range of somatic genetic alterations, with specific profiles for HPV-positive and HPV-negative tumors [7]. In addition, cancers of the various head- and neck anatomical sites are exposed to different environmental factors and have subsite-specific submucosae which may contribute to their difference in aggressiveness and response to treatment. The favorable outcome of HPV-positive oropharyngeal cancer was recently acknowledged in the eighth edition of the tumor-node-metastasis (TNM) staging system [8]. Thus, survival data from a pool of different head and neck cancer locations, as presented in the Pathology Atlas, should be interpreted with caution. Clinical–pathological variables such as gender, age, tumor stage, and tumor-to-stroma ratio may also affect patient survival, and these parameters can only be partly controlled for in the Pathology Atlas. Therefore, the data on prognostic factors for head and neck cancer listed in the Pathology Atlas need validation for the separate head and neck locations, where important clinical–pathological variables are adjusted for. Several studies have analyzed the publicly available TCGA HNSCC data set [7,9,10,11,12], but to the best of our knowledge, none have validated prognostic markers for HNSCC presented in the Pathology Atlas on both protein and gene expression levels.

In the present study, we aimed to validate some of the most promising prognostic factors for head and neck cancer from the Pathology Atlas in a cohort of oral tongue squamous cell carcinoma (OTSCC) patients, by both reverse transcription quantitative PCR (RT-qPCR) and immunohistochemical (IHC) analyses.

Two of the factors assessed showed prognostic value in univariate analyses, but only one of them was an independent prognostic factor in the OTSCC cohort. This highlights the need to evaluate prognostic value in homogenous groups of cancers controlling for established risk factors.

## 2. Materials and Methods

### 2.1. Patients and Material

In this retrospective study, we used data from the HNSCC cohort in the Pathology Atlas. These data are based on the publicly available database of 499 HNSCC patients included in the TCGA, which we named the TCGA-HNSCC cohort. The characteristics of this cohort are listed in Table 1 and are except from the tumor location data derived from the Pathology Atlas website. The tumor locations are derived from the TCGA website for the original TCGA-HNSCC cohort that included about 30 additional patients (*n* = 527).

To obtain an estimate of how many of the TCGA-HNSCC cohort that were HPV-negative oral SCC, we first excluded all patients with locations that were not potentially the oral cavity proper (larynx, tonsils, base of tongue, oropharynx, hypopharynx, and lip). For the remaining 318 patients, many were listed with unspecific locations that could include areas that belong to the oropharynx and possibly be HPV-positive tumors. The gene expression data of these patients were downloaded from the TCGA website, and assessed according to gene profiles published for HPV-positive HNSCC and HPV-negative HNSCC [7]. Based on these analyses, 157 patients of the TCGA-HNSCC cohort were HPV-negative oral or oropharyngeal SCC. We aimed at validating prognostic data from the TCGA-HNSCC cohort in 121 primary treatment-naïve oral tongue (OT) SCC, which we named the OTSCC cohort. The OTSCC cohort consisted of patients with SCC confined to the anterior two-thirds of the OT, diagnosed at the four head and neck cancer centers in Norway (the university hospitals of Oslo, Bergen, Trondheim, and Tromsø) from 1 January 2005 through 31 December 2009. The OTSCC cohort was collected in the retrospective Norwegian Oral Cancer (NOROC) study [13], where experienced head and neck surgeons collected relevant clinical data and TNM classification from the patients’ hospital files. For patients who underwent neck surgery (*n* = 84), the N status was based on histopathological analysis (pN). For all other patients (*n* = 38), the N status was based on clinical/radiological examination (cN). All tumors were reclassified by experienced pathologists in accordance with the eighth edition of the TNM classification [3], with the T status based on histopathological examination including tumor depth. The last day of follow-up was 1 June 2015, when all patients were followed up for a minimum of five years or until death. We retrieved the cause of death from the Cause of Death Registry if it was not reported in the patient’s files. Table 2 summarizes the clinical characteristics of the OTSCC cohort.

The patient information was deidentified prior to analysis. The study was approved by the Regional Ethics Committee of Northern Norway (2013/1786 and 2015/1381), which deemed it unnecessary to obtain written or oral consent from the participating patients, though they had the opportunity to opt out.

### 2.2. Tissue Microarray (TMA)

TMAs from formalin-fixed, paraffin-embedded (FFPE) tumor tissue blocks of the OTSCC cohort were constructed in a fully automated tissue microarray machine (TMA Master II, 3DHISTECH) as previously described [14]. In brief, two to four tissue cores with a diameter of 2 mm from the invasive front and more superficial parts of the tumors were arrayed into the recipient paraffin blocks.

### 2.3. Selection of Markers

We searched the Pathology Atlas’ lists of markers that were most significantly associated with survival for HNSCC. We selected genes that were most likely associated with tumor cells based on functions and expression profiles described in the HPA and in The National Centre for Biotechnology Information (NBCI) database. Additionally, the genes had to encode proteins that had HPA-validated antibodies for immunohistochemistry (IHC), and that according to data from the HPA had distinct expression patterns to promote reliable and reproducible scoring of the IHC staining. The reasoning behind the selection of markers is illustrated in the Supplementary File, Table S1. Based on this initial screening, the markers Calmodulin-like 5 (CALML5), LIM domain and actin-binding 1 (LIMA1), and CD59 were selected for validation of prognostic value of both protein and transcript (mRNA) in our OTSCC cohort. In the Pathology Atlas these were listed with the following 5-year overall survival data: CALML5 high 54% versus CALML5 low 37%, *p* = 0.000026; LIMA1 high 36% versus LIMA1 low 56%, *p* = 0.0000018; CD59 high 31% versus CD59 low 50%, *p* = 0.00031. Testing of the antibody staining is described below.

### 2.4. IHC Staining and Scoring

Four-μm-thick sections of the TMA blocks on Superfrost slides were deparaffinized in xylene and rehydrated in graded alcohol baths. Antibodies, antigen retrieval procedures, dilutions, and incubation times, as well as positive and negative controls, are listed in Table 3.

Prior to incubation with primary antibodies, the slides were incubated 30 min with 3% H_2_O_2_ to block endogenous peroxidase activity, and incubated one hour with 10% goat serum (Dako, Glostrup, Denmark) in phosphate buffered saline (PBS) (Dako, Glostrup, Denmark) to reduce unspecific staining. Bound primary antibodies were visualized using the anti-rabbit Envision Plus System (K4011, Dako, Glostrup, Denmark). The slides were washed in PBS after incubation with primary and secondary antibodies. All antibodies used had been thoroughly validated in the HPA project. In addition to the positive control tissues listed in Table 3, oral tissue from non-inflammatory fibrous hyperplasia of non-cancer patients as well as tumor sections from some patients were included to evaluate the staining.

The stained TMA-sections were scanned in an Olympus VS120 slide scanner (Olympus, Germany) and evaluated using the OlyVIA software version 1.06 (Olympus, Germany). Two independent, trained observers examined all cores. The observers were blinded to the clinical outcome of the patients. The cores were given a score based on the proportion of positive tumor cells: no staining (0), positive staining in less than 25% of the tumor cells (1), positive staining in 25–50% of the tumor cells (2) or staining in >50% of the tumor cells (3). Representative images of the different staining are presented in the Supplementary File, Figure S1. One of the observers analyzed the cores twice, and inter- and intra-observer variability in scoring was calculated. In the case of differing scores, agreement was reached by re-evaluating and discussing the staining together. We calculated a mean staining score for each patient with at least two evaluable cores, and dichotomized the patients into high expressers and low expressers based on this score and according to specific cut-off points. For each marker we tested the cut-off between high and low expressers at each quartile: 25% lowest vs. rest; 50% (median); and 75% highest vs. rest. We reported the results for the median as well as the quartile that gave the best separation of survival between the groups if this was not the median cut-off.

### 2.5. RNA Extraction and Quality Control

The prognostic values listed in the Pathology Atlas are based on transcriptional data. Thus, we also analyzed the prognostic value of the selected markers using RT-qPCR analyses. From cases with sufficient residual tumor material, we isolated total RNA from FFPE OTSCC tissue blocks using the AllPrep DNA/RNA FFPE kit from Qiagen (80234; Qiagen, Hilden, Germany). We sectioned 10-μm-thick sections from 65 of the FFPE OSCC tissue blocks, and put four consecutive sections of each patient onto glass slides. Slides were incubated for 1 h at 65 °C, then at 4 °C overnight before deparaffinization in xylene and rehydration in graded alcohol baths. We identified and marked areas with cancer tissue under the light microscope, carefully hydrated the sections with buffer PKD from the AllPrep DNA/RNA FFPE kit and scraped off the cancer tissue with a sterile scalpel into Eppendorf tubes containing 150 µL buffer PKD. Cancer tissue from four sections of each patient was collected into one tube, and the manufacturer’s protocol was followed from this step on. RNA was eluted by 25 μL RNase free water (20 μL for smaller tumor sections).

We measured total RNA quantity using the NanoDrop spectrophotometer (Thermo Scientific, Wilmington, DE, USA) and assessed RNA integrity number (RIN) using the Experion automated electrophoresis system (Bio-Rad Laboratories, Hercules, CA, USA). The RIN values ranged from one to four, which is as expected based on results from previous studies using RNA from FFPET [15].

### 2.6. Reverse Transcription Quantitative PCR (RT-qPCR)

We used the QuantiTect Reverse Transcription kit (Qiagen, Hilden, Germany) to reverse-transcribe 100–200 ng total RNA to cDNA, which was subsequently diluted 1:15 in nuclease-free water. RT-qPCR was performed in duplicates or triplicates using the Light Cycler 96 instrument (Roche, Mannheim, Germany). Target cDNA was amplified through 40 cycles in 20-µL reactions containing 1 × FastStart Essential DNA Green Master (Roche), 10 µL of diluted cDNA (1:15), and 300-nM primers. The primers used are listed in Table 4. As mRNA was extracted from FFPE tissue, we designed short primers to ensure that most of the available degraded RNA was amplified. 

The amplification efficiency for each gene was calculated from the slope and correlation coefficient (R2) of regression curves from 2-fold serially diluted cDNA (Table 4). Melting curve analysis was used to verify the specificity of the primers. Controls with the reverse transcriptase omitted and non-template controls were included to test for genomic DNA contamination and carry-over products. A positive control consisting of cDNA from three different fresh frozen lymphoid tissues was included in each run. The ∆∆Ct method [16] was used to calculate the relative amount of target mRNA normalized against the geometric mean of the reference genes elongation factor 1 alpha (eF1a), ribosomal protein L27 (RPL27), and ribosomal protein S13 (RPS13). These genes have earlier been identified as the most stable reference genes in a similar oral cavity cancer cohort [15].

### 2.7. Statistical Analysis

We used SPSS software version 22.0 for Windows (IBM, Armonk, NY, USA) and Microsoft Excel 2013 (Microsoft, Redmond, WA) for all calculations. Intra- and inter-observer variability for the IHC scoring was analyzed using the Spearman correlation test. We used univariate Kaplan–Meier analyses to calculate 5-year DSS rates, and the log-rank test to evaluate the statistical significance. Multivariate analyses were done using a stepwise forward multiple Cox regression model. Linear regression analyses of standard curves derived from serially diluted cDNA were used to estimate RT-qPCR amplification efficiency. The significance level was set to *p* < 0.05, with *p* < 0.1 being evaluated as borderline significant.

We followed the reporting recommendations for tumor marker prognostic studies (REMARK) to allow transparency and reproducibility of our prognostic marker studies [17,18].

## 3. Results

### 3.1. Immunohistochemical Staining and Scoring

The markers CALML5, CD59, and LIMA1 were chosen based on their promising prognostic value presented in the Pathology Atlas and their perceived cancer cell specificity, as well as the availability of validated antibodies. The antibodies also showed distinct staining patterns in our hands, with staining in positive controls as predicted from expression data. All negative controls were without staining. In the tumor tissue, the markers were only expressed in cancer cells, and with clear differences between patients. Evaluation of full tumor sections from selected patients showed reasonable staining homogeneity within a tumor. The staining also differed between cancer tissue and non-cancerous oral mucosa. We observed both membranous and intracellular staining of the respective markers. Representative images of scores 1, 2, and 3 of the various IHC stainings are shown in the Supplementary File, Figure S1. The inter- and intra-observer correlation was very good (r > 0.9) for scoring of CALML5 staining, and good (r > 0.75) for the CD59 and LIMA1 staining, confirming that the selected markers could be scored with high consistency.

### 3.2. Univariate Analyses

Of the clinical–pathological variables for the OTSCC cohort, N status, stage, tumor differentiation, and lymphocyte infiltration were significantly associated with 5-year DSS in univariate analyses (Table 2). None of the transcripts selected from the Pathology Atlas were significantly associated with 5-year DSS in the OTSCC cohort based on RT-qPCR analyses (Table 5). Kaplan–Meier curves are shown in the Supplementary File, Figure S2.

**Figure 1:**
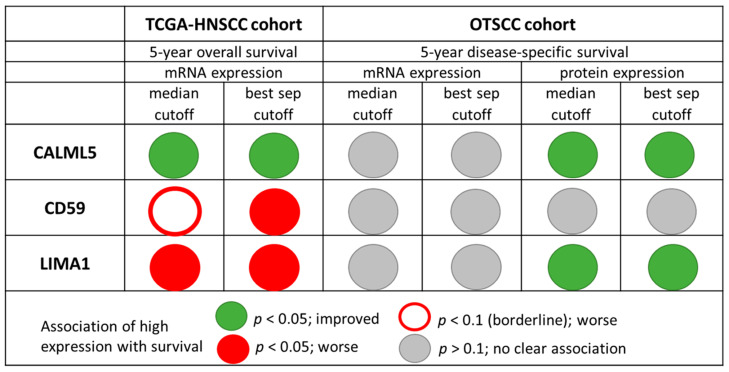
Association of high expression of CALML5, CD59, and LIMA1 mRNA and protein expression with 5-year overall survival in the TCGA head and neck cancer (TCGA-HNSCC) cohort and 5-year disease-specific survival in the oral tongue squamous cell carcinoma (OTSCC) cohort.

When analyzing the proteins encoded by these transcripts using IHC, high expression of CALML5 and LIMA1 were both significantly associated with increased 5-year DSS in univariate analyses (*p* = 0.016 and *p* = 0.043, respectively). For protein expression of CALML5 and LIMA1, the median cut-offs showed best survival separation. Of note, a high expression of the LIMA1 transcript was associated with decreased survival in the TCGA-HNSCC cohort (Figure 1), the opposite effect of what we found for high protein expression in the OTSCC cohort. Protein expression of CD59 showed no statistically significant association with 5-year DSS. In the OTSCC cohort, target protein and mRNA expression were significantly correlated (Spearman’s rank correlation) only for CALML 5 (r = 0.34, *p* = 0.017). For LIMA1, the correlation between target protein and mRNA was negative, although not statistically significant (r = −0.05, *p* = 0.680).

### 3.3. Multivariate Analyses

For the OTSCC cohort, Cox regression analyses with forced entry were performed for variables significantly associated with 5-year DSS in univariate analyses (N status, tumor differentiation, lymphocyte infiltration, CALML5 protein expression, and LIMA1 protein expression). The T status was also included in the models and dichotomized into T1 versus T2/T3. Separate analyses were run for LIMA1 and CALML5. All included variables fulfilled the proportional hazards assumption (Supplementary File, Figure S3).

N status, tumor differentiation, and CALML5 were significant, independent prognostic factors for 5-year DSS (Table 6).

## 4. Discussion

Identification of promising prognostic markers by so-called unbiased searches has become increasingly popular during the last decade. The Pathology Atlas has through an unbiased approach correlated transcriptional data from TCGA to overall survival in 17 major human cancers, and thereby made a substantial contribution to this field of research. In line with the concept of unbiased searches, the majority of the 793 prognostic markers for HNSCC in the Pathology Atlas have never been tested for prognostic value in such cancers previously, and many of them have poorly defined functions with low tissue or cell specificity.

In the present study, we sought to validate the prognostic value of three of these transcripts, LIMA1, CALML5, and CD59, in a homogenous cohort of OTSCC. In the TCGA-HNSCC cohort, gene expression of CALML5 was significantly associated with better survival, whereas CD59 and LIMA1 were significant predictors of worse survival. CALML5 is expressed in keratinocytes, and has an important role in epidermal differentiation [19]. Ubiquitination of CALML5 has been associated with breast carcinogenesis [20]. Furthermore, methylation of the CALML5 gene, which may repress transcription, was associated with poor survival for HPV-positive oropharyngeal cancer patients [21]. This is in line with our results showing that high protein expression of CALML5 is an independent predictor of longer survival in OTSCC patients, and suggests that this protein is as an interesting target for further research. LIMA1 has been described as an actin-binding protein that is involved in actin cytoskeleton regulation, and it is frequently lost in human solid cancers [22,23,24]. Recently, LIMA1 was identified as a direct transcriptional target of p53, and downregulation of LIMA1 caused by p53 mutation has been associated with poor survival of cancer patients, probably through initiating the invasion-metastasis cascade [25]. This is in line with our finding that high expression of LIMA1 at the protein level was associated with longer survival in OTSCC patients; however, it was not an independent prognostic marker. CD59 is a membrane complement regulatory protein that protects target cells from complement injury [26]. CD59 overexpression in HNSCC was mediated by the tumor microenvironment, and may be a mechanism to escape from complement attack [27]. In our OTSCC cohort, CD59 did not have any significant prognostic value at the protein level, and none of the selected markers had prognostic value at the mRNA level. 

There may be many reasons for the lack of coherence between our results and the data reported by the Pathology Atlas. HNSCC comprise many anatomical subsites, each with distinct presentation and behavior [28,29]. Most notable is the high prevalence of HPV-positive oropharyngeal cancers, which are associated with a better prognosis than HPV-negative cancers. Furthermore, the oropharynx differs from other head and neck sublocations by the predominance of lymphoid tissue. Thus, it is not surprising that the prognostic markers for HNSCC in the Pathology Atlas are not applicable to all head and neck subsites. Furthermore, the prognostic data in the Pathology Atlas are based on univariate analyses. We found that LIMA1 had prognostic value at protein level in univariate analyses, but not in multivariate analyses. Our study therefore highlights the need to validate the prognostic factors of the Pathology Atlas for specific anatomical subsites, and to adjust for known risk factors to identify independent prognosticators. We found tumor differentiation and N status, which are both well-recognized prognostic factors, to be the strongest independent prognosticators of those assessed in the OTSCC cohort.

Cancers of the oral mobile tongue as in our OTSCC cohort are typically HPV-negative [14]. When estimating the number of HPV-negative oral cancers in the TCGA-HNSCC cohort, we were left with 157 tumors, of which several were probably oropharyngeal cancers as the TCGA does not provide information on the exact tumor location. Thus, despite the discrepancy in number of patients included in the OTSCC and in the TCGA-HNSCC cohort (*n* = 121 and *n* = 499, respectively), the number of patients with HPV-negative oral cancers in the two cohorts was comparable. The OTSCC cohort, however, was much more homogenous and also had well-validated clinical and histopathological data from patients that were treated with curative intent only. Thus, we still argue that the data derived from this cohort are the most reliable for OTSCC. Yet, analyses on a larger OTSCC sample would be relevant to confirm the results, especially at the transcriptional level where lack of tumor tissue reduced the sample size in the present study.

For the OTSCC cohort, data on cause of death were available, which allowed survival analyses with disease-specific death as outcome. As these analyses censor patients dying of other reasons than the cancer, we believe that they give a more accurate estimation of the prognostic value of the assessed markers than overall survival. This is particularly relevant for cancers where the mean age at diagnosis is relatively high, such as for HNSCC, because this increases the risk of dying of other reasons than the disease during follow-up. The use of different endpoints in the HNSCC and the OTSCC survival analyses may have contributed to the differing results.

Direct comparison of results from gene and protein expression is difficult, as only a small fraction of the RNA will be translated to proteins. The remaining RNA is involved in complex, regulatory processes, which influence the production of proteins. Only recently, the strict classification as coding and non-coding transcripts has been questioned, as bi-functional RNAs with both coding and non-coding roles have been identified [30]. Regulation of coding and non-coding activity can be temporal, and some of our coding target transcripts may harbor non-coding, regulatory functions at specific stages during tumor development, which can affect protein synthesis and cellular function. The complex roles of the transcriptome could partly explain why we found different prognostic value of mRNA and protein of our selected markers. In our OTSCC cohort, CALML5 was the only marker where gene and protein expression were significantly correlated, and LIMA1 even showed a negative, but not significant correlation, indicating regulatory functions for some of our selected transcripts.

TCGA reports transcriptional data from tumor tissue, but information on where in the tumor and how the tissue for RNA extraction was selected is limited. As long as microdissection has not been performed, the samples will be a mixture of cancer cells and stromal cells, and the proportion of different cell types will vary dependent on the tumor’s growth pattern and from where in the tumor the sample is taken. Furthermore, the composition of the tumor stroma has important implications for the pathogenesis and prognosis of HNSCC [31,32], and may differ markedly between tumors. The survival analyses in the Pathology Atlas are based on the number of transcripts per patient sample, but the lack of knowledge of which cells have contributed to the transcripts is an important limitation of the method. In an effort to reduce the variation in tumor to stroma ratio between our samples, we placed thick tumor sections on histological glass-slides, and macro dissected out areas rich in cancer cells for RNA extraction. We further selected markers for validation that were associated with the cancer cells. Differences in the extraction procedures and the composition of samples for RNA extraction may have contributed to the lack of coherence between results from the Pathology Atlas and our study. Furthermore, we extracted mRNA from FFPE tissue which will inevitably be degraded. We used extraction procedures and reagents optimized for FFPE tissue, and designed primers with short amplicon length, which showed high amplification efficacy and consistency. However, differences in degradation status and RNA extraction methods may have contributed to the discrepancy in prognostic value between our and the Pathology Atlas cohort.

## 5. Conclusions

We found that high expression of CALML5 at protein level is an independent positive prognostic factor in OTSCC patients, and announced this protein as an interesting target for further research. The prognostic value of CD59 and LIMA1 reported in the Pathology Atlas could not be validated in our OTSCC cohort, neither at mRNA nor protein level. The well-established prognostic parameters, tumor differentiation and N status [33], were the strongest independent prognosticators in our cohort. Our findings illustrate that unbiased biomarker approaches can be valuable for identification of potential new prognostic markers. However, they also highlight the need for validation in homogenous patient cohorts, adjusting for known risk-factors. Almost 800 transcripts showed significant association with survival of HNSCC in the Pathology Atlas, and although we could only validate one of them in OTSCC, there are many candidates left to assess.

## Figures and Tables

**Table 1:** Clinical characteristics of the TCGA-HNSCC cohort.

The TCGA-HNSCC Cohort	*n* (%)
TCGA-HNSCC patients included in The Pathology Atlas analyses	499 (100)
**Gender**	
Male	366 (73)
Female	133 (27)
**Stage**	
I	25 (5)
II	69 (14)
III	78 (16)
IV	259 (52)
Information missing	68 (14)
**Alive at data collection**	
Yes	281 (56)
No	218 (44)
**Location (original TCGA-HNSCC cohort)**	**527 (100)**
Other and unspecified parts of tongue	132 (25)
Larynx	117 (22)
Other and ill-defined sites in lip, oral cavity, and pharynx	71 (13)
Floor of mouth	56 (11)
Tonsil	46 (9)
Other and unspecified parts of mouth	43 (8)
Base of tongue	24 (5)
Gum	11 (2)
Oropharynx	10 (2)
Hypopharynx	9 (2)
Palate	5 (1)
Lip	3 (1)

**Table 2:** Clinical–pathological characteristics of oral tongue squamous cell carcinoma patients with survival data (*n* = 121), and their association with 5-year disease-specific survival (DSS) in Kaplan–Meier analysis.

Variable		*n*	5-year DSS %	*p*-Value ^1^
Gender	Male	75	66.7	0.789
	Female	46	69.6	
Age at diagnosis, years	<65	60	66.7	0.776
	≥65	61	68.9	
Smoking	Never	30	73.3	0.521
	Current	51	66.7	
	Former	29	58.6	
	Missing	11	-	
T status	T1	37	83.8	0.072
	T2	47	61.7	
	T3	29	65.5	
	Unknown	8	-	
N status ^2^	N0	84	81.0	<0.001
	N+	36	36.1	
	Missing	1	-	
Stage	Low stage (stage I or II)	62	82.3	<0.001
	High stage (stage III or IV)	55	50.9	
	Nx/Unknown	4	-	
Differentiation, whole tumor	Low-grade (well or moderate)	106	73.6	<0.001
	High-grade (poor)	13	23.1	
	Missing	2	-	
Lymphocyte infiltration	Abundant	77	74.0	0.029
	Little	38	55.3	
	Missing	6	-	

^1^ The *p*-value was calculated using the log-rank test, with the missing/unknown cases for the respective variables omitted, and the significance level set to 0.05 ^2^ Combination of cN and pN. In case of neck dissection, the result on pN was superior to cN.

**Table 3:** List of antibodies and immunohistochemical procedure.

Antibody	Antigen Retrieval	Blocking	Wash Buffer	Dilution	Incubation Time and Condition	Secondary Antibody	Positive Control
Anti-CD59, HPA026494, Sigma–Aldrich	Citrate buffer pH 6.0	1.5% goat serum (Dako X9070)	PBS	1:100	30 min room temperature	Anti-rabbit HRP conjugated (Dako K-4011)	Human tonsils
Anti-CALML5, HPA040725, Sigma–Aldrich	Citrate buffer pH 6.0	1.5% goat serum (Dako X9070)	PBS	1:2000	Overnight 4 °C	Anti-rabbit HRP conjugated (Dako K-4011)	Hum. salivary glands
Anti-LIMA1, HPA052645, Sigma–Aldrich	Citrate buffer pH 6.0II	1.5% goat serum (Dako X9070)	PBS	1:200	Overnight 4 °C	Anti-rabbit HRP conjugated (Dako K-4011)	Human intestine

**Table 4:** Primers for RT-qPCR analyses.

Gene	Accession No.	Full Name	Primer Sequence (5′ to 3′)	Ampl/effic/corr	Size (bp)
**Reference RNA**					
eF1a	NM001402.5	Elongation factor 1 alpha	F: TATCCACCTTTGGGTCGCTTT	99.8/1.000	63
			R: TGATGACACCCACCGCAACT		
RPL27	NM000988.3	Ribosomal protein L27	F: GCTGGACGCTACTCCGGAC	96.8/0.998	64
			R: CGATCTGAGGTGCCATCATCA		
RPS13	NM001017.2	Ribosomal protein S13	F: AGAGAGCCGGATTCACCGTTT	95.1/0.999	62
			R: CAATTGGGAGGGAGGACTCG		
**Target RNA**					
CALML5	NM017422.4	Calmodulin-like 5	F: CGGTGAGCTGACTCCTGAGG	97.3/0.999	84
			R: GGCATTGATGGTGCCGTTT		
CD59	NM000611.5; NM001127223.1; NM001127225.1; NM001127226.1; NM001127227.1; NM203329.2; NM203330.2; NM203331.2	CD59	F: GGGTGTCAGTCAGGGACAACA	98.3/0.999	92
			R: TTCATGCCCTGCTATCTGGA		
LIMA1	NM001113546.1; NM001113547.1; NM001243775.1; NM016357.4	LIM domain and actin-binding 1	F: GCCAAGGCCTCCTCTCAGC	101.9/0.999	68
			R: CCAGGCGATCCTCAGCTTCT		

Ampl effic = amplification efficiency, corr = correlation coefficient for 2-fold serially diluted cDNA.

**Table 5:** Target protein and mRNA expression in the oral tongue squamous cell carcinoma cohort using median and best separation cut-off, and their association with 5-year disease-specific survival (DSS) in Kaplan–Meier analysis. The *p*-value was calculated using the log-rank test, with the significance level set to 0.05.

		Oral Tongue Squamous Cell Carcinoma (OTSCC) Cohort											
		mRNA Expression						Protein Expression					
		Median Cut-off			Best Separation Cut-off			Median Cut-off			Best Separation Cut-off		
		*n*	DSS %	*p*-Value	*n*	DSS %	*p*-Value	*n*	DSS %	*p*-Value	*n*	DSS %	*p*-Value
CD59	Low	36	69.4	0.256	= median			44	61.4	0.493	18	55.6	0.398 ^1^
	High	28	57.1					74	70.3		100	69.0	
CALML5	Low	23	52.2	0.192	= median			66	57.6	0.016	=median		
	High	24	75.0					52	78.8				
LIMA1	Low	39	69.2	0.214	= median			52	57.7	0.043	=median		
	High	25	56.0					64	75.0				

^1^ The best separation cut-off for CD59 protein expression was 25%.

**Table 6:** Multivariate analysis of 5-year disease-specific survival in the oral tongue squamous cell carcinoma (OTSCC) cohort in accordance with Cox’s proportional hazards model. N status, tumor differentiation, T status, and lymphocyte infiltration were adjusted for CALML5 and LIMA1 separately. Only patients with data for all respective variables were included (*n* = 105 and *n* = 103 for CALML5 and LIMA1, respectively).

	Adjusted for CALML5			Adjusted for LIMA1		
Variable *n* (CALML5/LIMA1)	Hazard Ratio	95% CI	*p*-Value	Hazard Ratio	95% CI	*p*-Value
N status N0, *n* = 75/74 vs. N+, *n* = 30/29	0.337	0.159–0.714	0.005	0.302	0.143–0.636	0.002
Differentiation, whole tumor Low-grade (well or moderate), *n* = 93/92 vs. high-grade (poor), *n* = 12/11	0.389	0.172–0.879	0.023	0.227	0.114–0.673	0.005
T status T1, *n* = 32/32 vs. T2/T3, *n* = 73/71	0.458	0.177–1.184	0.107	0.508	0.201–1.288	0.154
Lymphocyte infiltration Abundant, *n* = 69/68 vs. little, *n* = 36/35	0.529	0.262–1.067	0.075	0.738	0.331–1.644	0.457
CALML5 protein expression Low, *n* = 57 vs. high, *n* = 48	2.363	1.011–5.523	0.047			
LIMA1 protein expression Low, *n* = 46 vs. high, *n* = 57				1.834	0.857–3.926	0.118

## Data Availability

The data presented in this study are available on request from the corresponding author. The data are not publicly available due to EU GDPR regulation.

## Supplementary Materials

The following are available online at https://www.mdpi.com/article/10.3390/cancers13102387/s1, Figure S1: Representative immunohistochemical staining and scoring for CALML5, CD59, and LIMA1, Figure S2: Kaplan-Meyer curves for 5-year disease-specific survival. Figure S3: Log minus log plots for proportional hazards checking. Table S1: Reasoning behind selection of prognostic markers for Head and neck cancer in the Pathology Atlas to validate in a cohort of oral tongue cancer.
