# Supplementary material for: Validation of Selected Head and Neck Cancer Prognostic Markers from the Pathology Atlas in an Oral Tongue Cancer Cohort
Source: Cancers (Basel). 2021 May 14;13(10):2387. doi: 10.3390/cancers13102387 (PMC8156750; doi:10.3390/cancers13102387)
Supplement: Supplementary file 1 [file cancers-13-02387-s001.zip › cancers-1200046-supplementary.pdf]

*Supplementary Materials*

# Validation of Selected Head and Neck Cancer Prognostic Markers from the Pathology Atlas in an Oral Tongue Cancer Cohort

**Anna Maria Wirsing [1], Inger-Heidi Bjerkli [1,2], Sonja Eriksson Steigen [1,3], Oddveig Rikardsen [1,2], Synnøve Norvoll Magnussen [1], Beate Hegge [1], Marit Seppola [1], Lars Uhlin-Hansen [1,3] and Elin Hadler-Olsen [1,4,*]**

[1] Department of Medical Biology, Faculty of Health Sciences, UiT The Arctic University of Norway, 9037 Tromsø, Norway; anna.wirsing@uit.no (A.M.W.); Inger-heidi.bjerkli@unn.no (I.-H.B.); sonja.eriksson.steigen@unn.no (S.E.S.); oddveig.rikardsen@unn.no (O.R.); synnove.magnussen@uit.no (S.N.M.); beate.hegge@uit.no (B.H.); Marit.seppola@uit.no (M.S.); Lars.uhlin-hansen@uit.no (L.U.-H.)

[2] Department of Otorhinolaryngology, University Hospital of North Norway, 9038 Tromsø, Norway

[3] Department of Clinical Pathology, University Hospital of North Norway, 9038 Tromsø, Norway

[4] The Public Dental Health Service Competence Centre of Northern Norway, 9019 Tromsø, Norway

* Correspondence: elin.hadler-olsen@uit.no; Tel.: +47-48-06-72-49

**Supplementary figures**

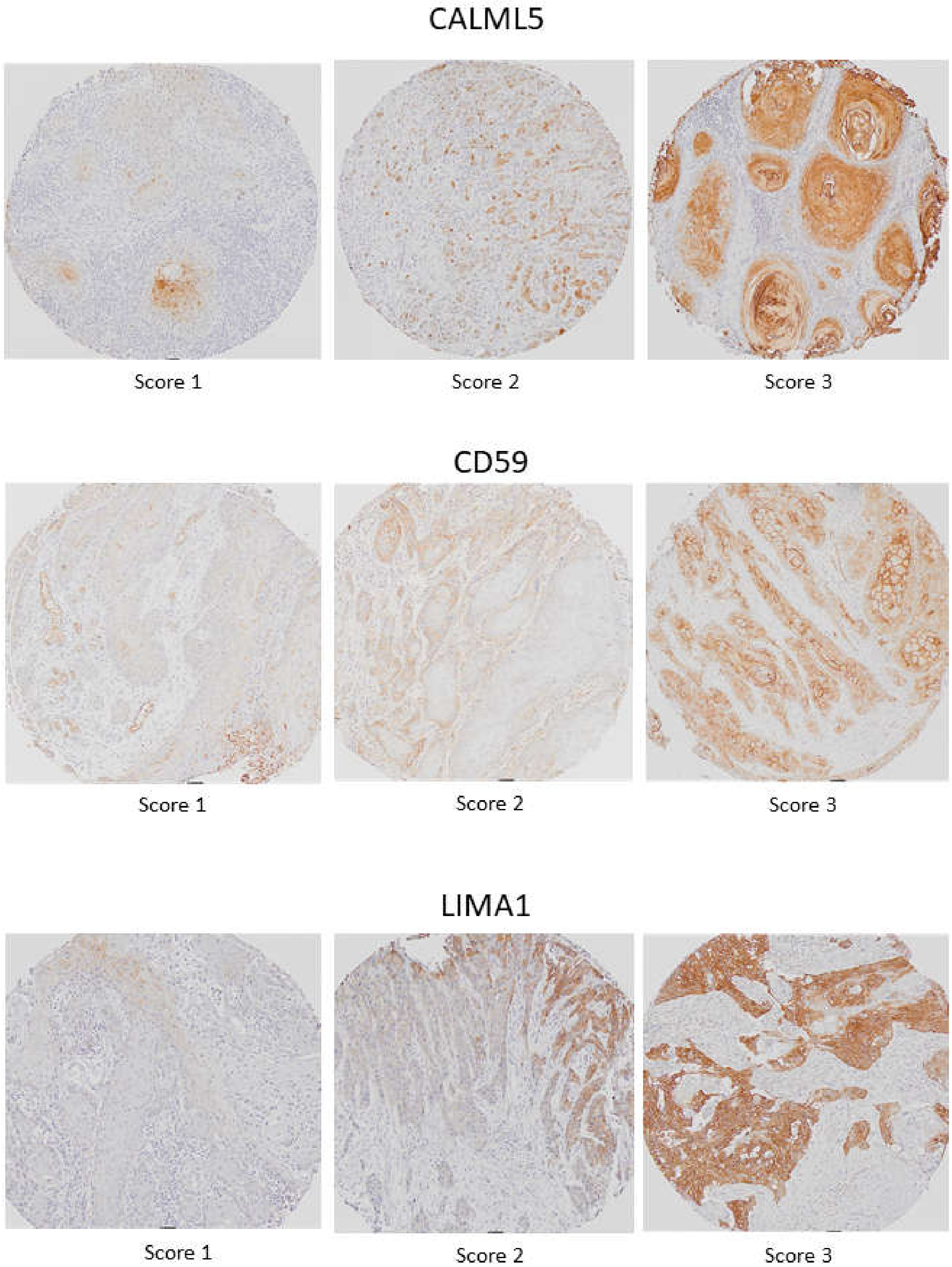


**Figure S1.** Representative immunohistochemical staining and scoring for CALML5, CD59 and LIMA1. Scores were assigned based on proportion of positively stained tumour cells: no staining (0), positive staining in less than 25% of the tumour cells (1), positive staining in 25-50 % of the tumour cells (2) or staining in > 50% of the tumour cells (3).

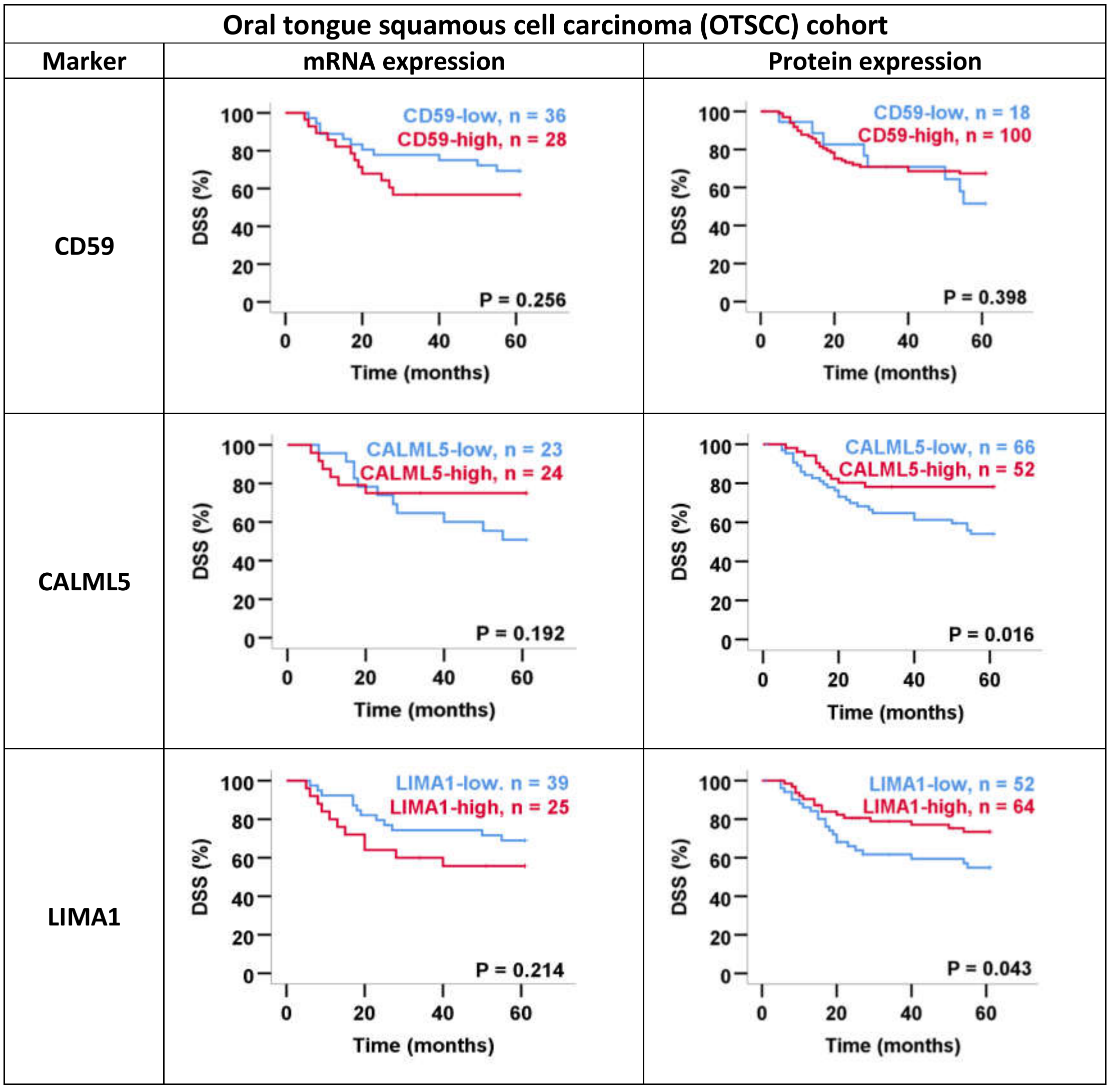


**Figure S2.** Kaplan-Meyer curves for 5-year disease-specific survival. Target protein and mRNA expression in the oral tongue squamous cell carcinoma cohort using best separation cutoff (median for all except CD59 protein, which was 25%), and their association with 5-year disease-specific survival (DSS) in Kaplan-Meier analysis. The p-value was calculated using the log-rank test, with the significance level set to 0.05.

cancers

MDPI

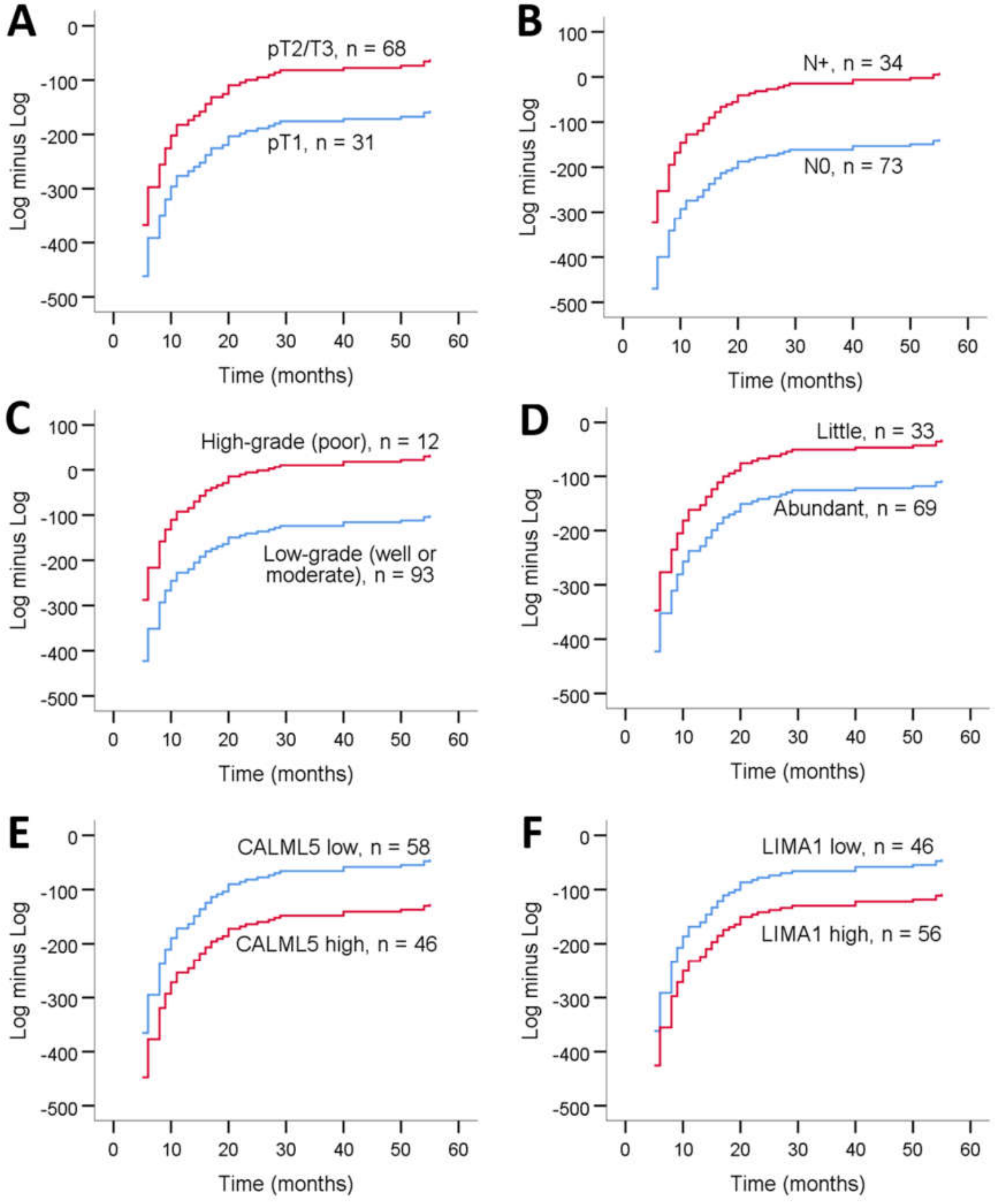


**Figure S3.** Log minus log plots for proportional hazards checking. A) pT status, B) N status, C) differentiation of whole tumour, D) lymphocyte infiltration, E) CALML5 protein expression, F) LIMA1 protein expression.

## Supplementary tables

**Table S1.** Reasoning behind selection of prognostic markers for Head and neck cancer in the Pathology Atlas to validate in a cohort of oral tongue cancer.

| Marker | Associated with tumour cells | HPA-validated antibody available | Expression pattern IHC | Conclusion |
|---|---|---|---|---|
| DKK1 | Unknown | No | No information | Excluded |
| LIMA1 | Yes | Yes | Intracellular | Included |
| CD59 | Yes | Yes | Intracellular, membrane | Included |
| GZMM | No | Yes | Intracellular, secreted | Excluded |
| CCR7 | No | Yes | Membrane | Excluded |
| CALML5 | Yes | Yes | Intracellular | Included |
